# Lysosomal acid lipase promotes endothelial proliferation in cold-activated adipose tissue

**DOI:** 10.1080/21623945.2021.2013416

**Published:** 2021-12-25

**Authors:** Alexander W. Fischer, Michelle Y. Jaeckstein, Joerg Heeren

**Affiliations:** Department of Biochemistry and Molecular Cell Biology, University Medical Center Hamburg-Eppendorf, Hamburg, Germany

**Keywords:** Adipose tissue, thermogenesis, lipoprotein, triglyceride, endothelial cells, browning, proliferation, lysosome, lysosomal acid lipase

## Abstract

Oxidative tissues such as brown adipose tissue and muscle internalize large amounts of circulating lipids and glucose as energy source. Endothelial cells (ECs) provide a platform for regulated transport and processing of blood-borne nutrients. Next to this role, it has become recognized that intercellular crosstalk between ECs and underlying parenchymal cells is indispensable for maintenance of tissue homoeostasis. Here, we comment on our recent observation that capillary ECs in thermogenic adipose tissues take up and metabolize entire triglyceride-rich lipoprotein (TRL) particles in response to cold exposure. This process is dependent on CD36, lipoprotein lipase (LPL) and lysosomal acid lipase (LAL). Remarkably, loss of LAL specifically in endothelial cells results in impaired endothelial proliferation and diminished thermogenic adaptation. Mechanistically, cell culture experiments indicate that LAL-mediated TRL processing leads to the generation of reactive oxygen species, which in turn activate hypoxia-induced factor (HIF)-mediated proliferative responses. In the current manuscript, we provide *in vivo* evidence that LAL-deficiency impairs proliferation of endothelial cells in thermogenic adipose tissue. In addition, we show uptake of nanoparticle-labelled TRL and LAL expression in cardiac endothelial cells, suggesting a physiological function of endothelial lipoprotein processing not only in thermogenic adipose tissue but also in cardiac muscle.

## Thermogenic adipose tissue endothelial cells – controlling the fire

Brown adipocytes residing in brown adipose tissue (BAT) contribute to whole body energy expenditure and maintenance of euthermia. In addition, sustained cold exposure results in browning in white adipose tissues (WAT), a process characterized by the appearance of thermogenically active beige/brite adipocytes. For heat production, thermogenic brown and beige/brite adipocytes uncouple the electron transportation chain from oxidative phosphorylation using a unique mitochondrial protein, the uncoupling protein 1 (UCP1). Due to their high capacity for energy combustion, brown and beige/brite adipocytes can beneficially affect metabolic health in rodents and potentially in humans [1]. Thermogenic adipocytes internalize substantial amounts of fuels – mainly lipids and glucose – to maintain tissue function and replenish intracellular energy stores [2]. To ensure sufficient nutrient and oxygen supply, thermogenic adipose tissues are highly vascularized. In adipose tissue, the amount and density of ECs is crucial for tissue expansion and thermogenic adipose tissue browning [3–6]. The continuous capillary endothelium forms a tight barrier between the circulation and parenchymal adipocytes, creating the first contact site for circulating nutrients. Therefore, fuelling of underlying cells requires efficient uptake, processing and potentially transendothelial transport of lipids and glucose by ECs. ECs express a variety of transporters, receptors, and lipases to take up and process nutrients. The shuttling of lipids to parenchymal cells of BAT is facilitated by a multistep process, in which the EC membrane acts as a platform for LPL-mediated processing of TRL. Subsequently, the liberated fatty acids (FAs) are taken up by transport proteins such as CD36 or FATP [7–9]. In the classical view, the remaining TRL-remnants detach from the vascular endothelium, reach the liver via the circulation and are ultimately internalized by hepatocytes through lipoprotein receptor mediated endocytosis [10]. Notably and in contrast to this classical pathway, substantial amounts of whole TRL remnant particles are cleared by activated thermogenic BAT and WAT [2,11–13], yet the physiological significance and regulation of whole-lipoprotein particle uptake remained elusive. Another source for FAs are intracellular triglycerides (TGs) stored in lipid droplets (LDs). In brown adipose tissue, cold exposure induces the hydrolysis of stored TGs by activation of lipases such as adipose triglyceride lipase (ATGL) and hormone-sensitive lipase (HSL) [14]. BAT lipolysis was considered to be essential for regular thermogenic capacity and cold adaptation. In fact, whole body as well as adipose tissue-specific ATGL-deficiency in mice leads to impaired non-shivering thermogenesis [15,16]. However, recent studies showed that mice lacking ATGL or its co-activator *comparative gene identification-58* (CGI-58) specifically in brown adipocytes displayed regular thermogenic function [17,18]. Similarly, HSL-deficient mice are not cold sensitive [19,20]. By brown adipocyte-specific deletion of acyl CoA:diacylglycerol acyltransferase (DGAT) enzymes, it was even shown recently, that TG storage in LDs generally is not required for regular non-shivering thermogenic adaptation [21]. These data suggest that lipolysis of intracellular TG stores in brown adipocytes is dispensable for BAT-mediated cold-adaptation, as long as WAT-derived/circulating lipids or other fuels are available. This underlines an important role of whole TRL uptake in nutrient supply for thermogenic adipose tissues.

## Lysosomal lipoprotein processing in endothelial cells determines thermogenic capacity of adipose tissue

Using nanoparticle labelling we recently discovered that entire TRL particles colocalize with endothelial cells in activated BAT [22], pointing towards an active and regulated uptake process into these cells. Indeed, electron microscopy confirmed a sequential pathway, starting with initial attachment of TRL particles to the endothelial wall, followed by shrinkage and subsequently the appearance of the particles in intracellular vesicular organelles [22]. These organelles were biochemically identified as representing the endosomal/lysosomal compartment [22]. We thus hypothesized that entire TRL particles are hydrolysed in lysosomes to release lipid species that could in turn affect the metabolic activity of the underlying thermogenic adipocytes. The main triglyceride and cholesterol ester-hydrolysing enzyme in lysosomes is LAL [23]. Magnetic isolation of endothelial cells confirmed high expression levels of LAL in endothelial cells of both BAT as well as WAT [22]. Expression of LAL was found to be higher in ECs than in adipocytes and it was induced by cold activation, strengthening the hypothesis that endothelial LAL exerts an important physiological role in thermogenic adipose tissue. Another recent report described the uptake of chylomicrons into aortic ECs, in which the particles are internalized by ECs and subsequently hydrolysed in lysosomes under pathophysiological conditions. The authors of this paper propose a mechanism that contributes to lipid accumulation in ECs and co-cultured macrophages of mice lacking LPL [24]. However, in contrast to our report this paper found CD36 and LPL to be dispensable for chylomicron uptake, and instead described a pathway involving the receptor SR-BI, a lipoprotein receptor best known for mediating transendothelial transport of high-density lipoproteins (HDL) into liver and adrenals [25–28]. Generally, it is believed that EC forming large vessels are displaying different gene expression patterns than ECs lining small tissue-embedded capillaries [29,30]. In contrast to macrovascular VCAM1-positive ECs, microvascular cells express CD36, LPL and GPIHBP1 [29–32]. Accordingly, differences in endothelial TRL uptake mechanisms are not surprising but might be necessary for homoeostatic tissue function. Both pathways, the one described in [24] as well as in our recent report [22] converge at the lysosome and imply an important functional role for lysosomal lipid processing.

We therefore generated inducible endothelial cell-specific LAL knockout mice and examined thermogenic adipose tissue function in these mice, which in response to cold exposure was significantly reduced [22]. Depending on the duration of the acclimation protocol, we found both BAT as well as WAT thermogenic capacity to be strongly decreased. In addition to insufficient uptake of triglycerides as well as glucose, we observed lower levels of thermogenic marker expression as well as reduced appearance of beige/brite adipocytes. These findings implied a functional relationship between endothelial lipoprotein processing and the differentiation of the underlying parenchyma. Cold-induced recruitment of brown and beige/brite adipose tissues is accompanied by a strong induction of angiogenesis, the formation and sprouting of new blood vessels [6]. We thus examined more closely the vascular bed in thermogenic adipose tissues of LAL-deficient mice in response to cold exposure. We found the expression of endothelial marker genes and proteins as well as vascular density to be significantly reduced [22]. In line with this finding, we observed a strong induction of proliferation of cultured human HUVEC endothelial cells and adipocyte precursor cells *in vitro* following incubation with human TRL particles, an effect that was dependent on LAL action [22]. Furthermore, we found that this effect was due to an activation of the HIF1a transcription factor through a beta oxidation-dependent stimulation of ROS production following TRL uptake. This TRL-LAL-ROS-HIF axis most likely contributes to the lipoprotein-mediated stimulation of proliferation.

## LAL-deficiency impairs endothelial proliferation *in vivo*

**Figure 1.**
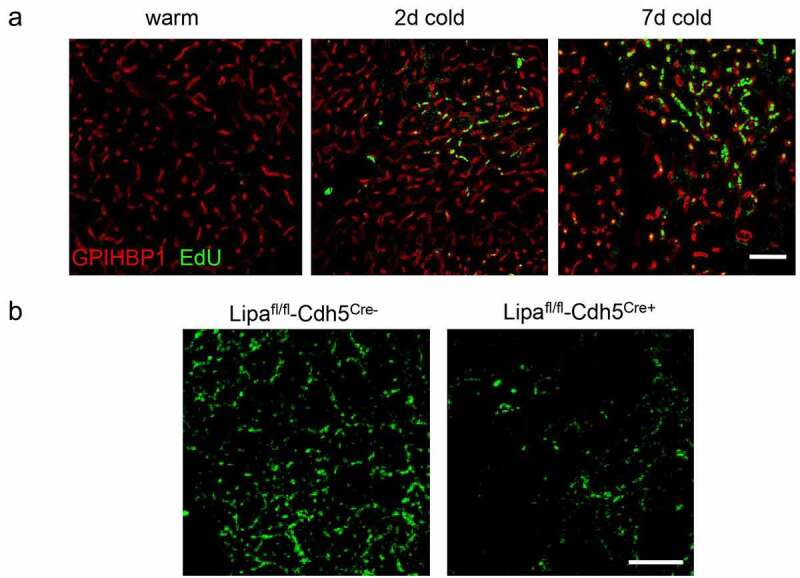
Endothelial cells proliferate in active BAT in a LAL-dependent manner

In our recent paper, the question remained as to whether the proliferative effect of LAL-mediated TRL-processing in ECs is also relevant *in vivo*. Here we investigated endothelial cell proliferation in a dynamic manner during cold exposure using *in vivo* incorporation of the thymidine analogue EdU (Figure 1(a)). Indeed, cold adaptation resulted in increased proliferation of EC. Moreover, in mice lacking LAL in endothelial cells we directly demonstrate that the proliferative responses in BAT endothelial cells were blunted *in vivo* (Figure 1(b)). In line with our published data, we confirmed that LAL-mediated TRL processing is required for regular cold-induced endothelial proliferation. However, the direct link between endothelial proliferation and increased thermogenic recruitment is not fully understood. It is conceivable that endothelial proliferation and activity directly signals to adipocyte precursor cells residing in the vascular niche in mice [33,34] and humans [35]. Moreover, endothelial cells have been shown to transdifferentiate into thermogenic adipocytes, providing another direct link between endothelial cell proliferation and the thermogenic recruitment of BAT and beige/brite WAT [5]. Further lineage tracing and single-cell sequencing experiments will be needed to decipher which pathway is affected by LAL deficiency and how the endothelial lipid state is sensed by precursor cells residing in the same niche.

(a) C57BL6/J wildtype mice were housed for the indicated times at 30°C or 6°C and received intraperitoneal injections of EdU (100 µg/g bodyweight) every other day. Following transcardial perfusion with 4% PFA in PBS, organs were fixed in formalin and embedded in paraffin. Rehydration and antigen retrieval was performed as described previously [39]. EdU was fluorescently labelled with Alexa594 using a Click-it EdU Imaging Kit (Invitrogen) according to manufacturer’s instructions, and tissues were co-stained with rat-anti-GPIBP1 antibody as described previously [22]. Representative images of EdU and GPIHBP1 co-staining in BAT are shown. Green = EdU, Red = GPIHBP1. Scale bar, 25 µm.(b) Lipa^fl/fl^-Cdh5^Cre^ mice were acclimated to 30°C for 2 weeks, followed by 3 days at 18°C and 4 days at 6°C. After 24 h of cold exposure, EdU treatment was initiated, and staining was performed as described above. Representative images of EdU staining in BAT are shown. Green = EdU. Scale bar, 25 µm.

## Future directions

**Figure 2.**
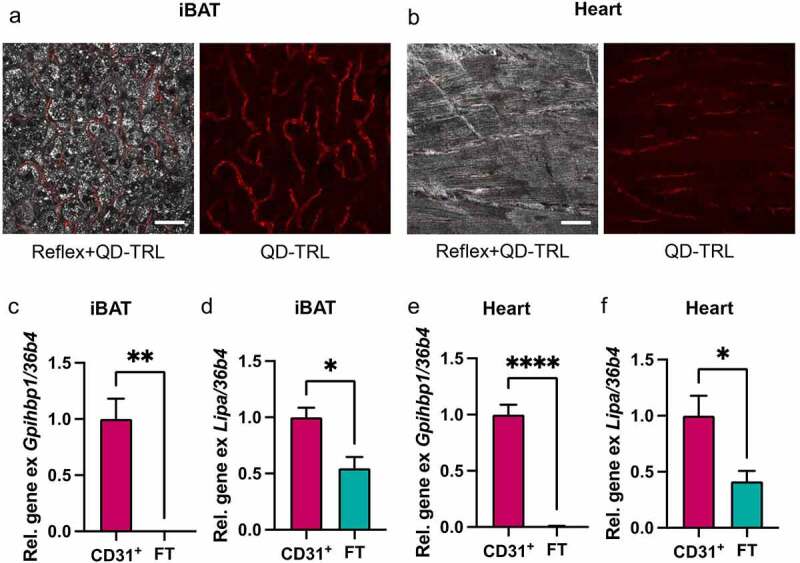
Endothelial cells of BAT and cardiac muscle take up whole TRL particles, and ECs are equipped to process TRLs in lysosomes

We identified lysosomal TRL processing by endothelial cells as an important process for efficient activation and thermogenic adaptation of BAT and WAT. However, many other oxidative tissues also process TRLs to ensure sufficient fatty acid supply. Muscle tissues are highly vascularized and endothelial cells from these tissues show signatures of high nutrient clearance capacity [36,37]. This is illustrated by the high expression levels of lipid metabolism players such as GPIHBP1, LPL and CD36 in cardiac and skeletal muscles. Given that the uptake of entire TRL particles in BAT/WAT depends on LPL and CD36, it appeared likely that similar pathways might also be present in the skeletal and cardiac muscles. In our recent publication, it remained elusive whether ECs localized in other tissues than BAT/WAT contribute to reduced TRL particle uptake and impaired thermogenic capacity in endothelial-specific LAL-deficient mice [22]. Here, using confocal microscopy following injection of QuantumDot-labelled TRL, we found the uptake of entire TRL particles both into endothelial cells of BAT (Figure 2(a)) and heart (Figure 2(b)). Furthermore, magnetic cell isolation experiments showed high enrichment of *Gpihbp1* in CD31-positive fractions of BAT (Figure 2(c)) and heart (Figure 2(e)). Notably, expression of LAL-encoding *Lipa* mRNA was significantly higher in endothelial versus parenchymal cells isolated from BAT (Figure 2(d)) and heart (Figure 2(f)). While we did not find any obvious signs of cardiac impairment in endothelial cell-specific LAL knockout mice, their lower total energy expenditure and body temperature upon cold exposure could point towards metabolic abnormalities beyond BAT and beige/brite WAT function [22]. Nevertheless, we found glucose and lipid uptake into heart and skeletal muscle to be unchanged in the endothelial cell-specific LAL knockout mice under conditions of warm or cold housing (unpublished results). Further studies using exercise regimens or models of cardiac impairment such as ischaemia-reperfusion may be used to shed light on the importance of TRL processing by endothelial LAL for (cardiac) muscle function.

C57BL6/J wildtype mice were injected with recombinant human TRL particles labelled with fluorescent Quantum Dot nanoparticles as described previously [11,22]. 20 min after intravenous injection, organs (BAT, cold activated or heart, standard housing temperature), tissues were dissected imaged as described previously [22,40].(a, b) Confocal microscopy images of QD-labelled TRL particles internalized by BAT (a) and heart tissue (B). Red = QD-TRL. Scale bar = 50 µm.Endothelial cells were isolated from BAT and heart as described previously [12] with minor alterations. Isolation of BAT EC was performed as described, while digestion of heart tissue was performed by incubation of finely minced tissue in PBS containing 10 mM CaCl2, 0.1% (w/v) collagenase I, 0.25% (w/v) collagenase IV, 2.4 U/mL Dispase and 7.5 μg/mL DNAse I for 45 min at 37°C with vigorous shaking and pipetting every 5 min. RNA extraction and qPCR was performed as described previously [22].(c, e) Gene expression of GPIHBP1 and (d, f) Lipa in CD31^+^ endothelial cells of BAT (c, d) or heart (e, f) isolated by MACS® (n = 4).

Furthermore, it remains to be established whether such pathway would be present and physiologically relevant in humans. Different levels of LAL deficiency result in distinct clinical manifestations [38]. Direct analysis of transcriptional and functional changes in ECs of oxidative tissues from patients presenting with LAL-associated diseases may provide insight into the relevance of this pathway in the human disease context.

## Data Availability

The data that support the findings of this study are openly available (DOI: 10.17632/xk9dtc7rw8.1) at Mendeley (https://data.mendeley.com).
